# Avulsion of the Ossification Center of the Iliac Crest in a Sporty Teenager: Multimodal Imaging Approach with Emphasis on the Role of the oZTEo MRI Pseudo-CT Sequence

**DOI:** 10.5334/jbsr.3542

**Published:** 2024-04-22

**Authors:** Quentin Rahier, Géraldine Debehogne, Frédéric E. Lecouvet

**Affiliations:** 1 Department of Radiology, Institut de Recherche Expérimentale et Clinique (IREC), Cliniques Universitaires Saint Luc, Université Catholique de Louvain, Brussels, Belgium; 2 Department of Radiology, Institut de Recherche Expérimentale et Clinique (IREC), Cliniques Universitaires Saint Luc, Université Catholique de Louvain, Brussels, Belgium; 3 Department of Radiology, Institut de Recherche Expérimentale et Clinique (IREC), Cliniques Universitaires Saint Luc, Université Catholique de Louvain, Brussels, Belgium

**Keywords:** apophysis, avulsion, sport injuries, MRI, ZTE sequence, zero echo time

## Abstract

*Teaching point:* The appearance of an avulsion of the ossification center of the iliac crest is reported on ultrasound, radiographs, and magnetic resonance imaging (MRI), with emphasis on the role of the “pseudo-CT” zero echo time (oZTEo) sequence to highlight the lesion.

## Case History

**Figure 1:**
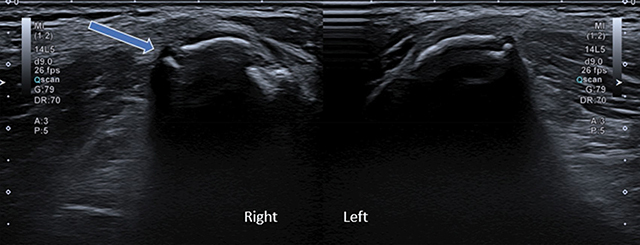
Ultrasound shows an irregular shape and displacement of the ossification center of the right iliac crest.

A 16-year-old male was referred for intense pain in his right hip area that appeared after an intense football training session. An ultrasound study was carried out 8 days after the onset of pain, which highlighted an irregular shape and slight lateral displacement of the ossification center of the right iliac crest compared to the left side (Figure 1, arrow).

**Figure 2:**
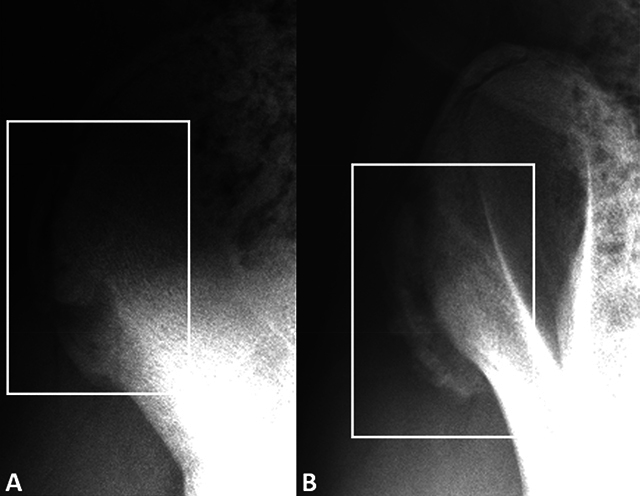
Radiograph of the right hip shows fragmentation and displacement of the anterior third of the ossification center of the iliac crest with periosteal reaction.

The radiographs performed 4 days later revealed fragmentation and displacement of the anterior third of the ossification center of the right iliac crest (Figure 2A) and adjacent periosteal reaction (Figure 2B).

**Figure 3:**
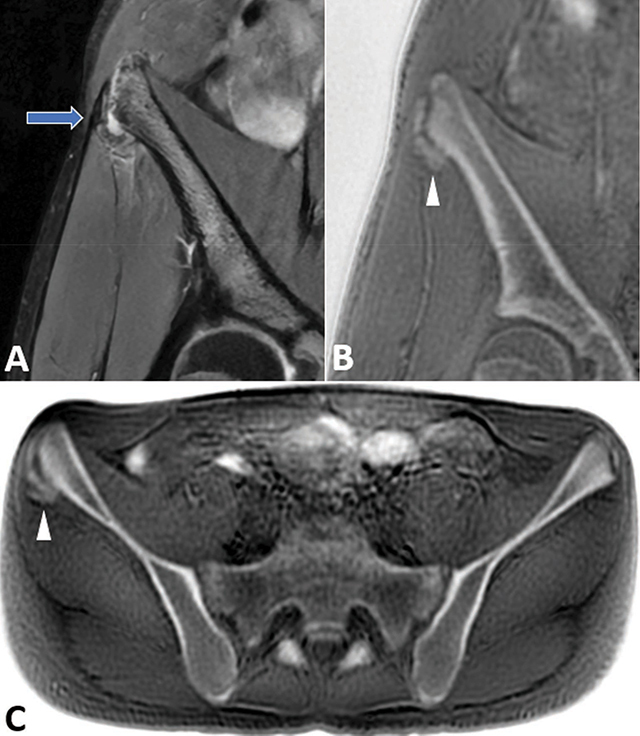
Coronal PDFS MR image shows lateral avulsion of the ossification center (**A**, arrow). Coronal and axial oZTEo images show the osseous displaced fragment with periosteal reaction (**B-C**, arrowhead).

A magnetic resonance imaging (MRI) study revealed a lateral avulsion of the ossification center. A coronal proton-density fat-saturated sequence showed a linear fluid-like area separating the nucleus from the crest (Figure 3A, arrow). Coronal (Figure 3B) and axial (Figure 3C) zero echo time (oZTEo) sequences confirmed the osseous/calcified nature of the avulsed fragment. The inverted grayscale windowing on these sequences provides a computed tomography (CT)-like image that enhances the lesion and adjacent periosteal reaction (Figures 3B–C, arrowhead).

## Comments

Apophyseal avulsions of the pelvis mainly affect adolescent athletes, with an average age of 14 years. Most of these avulsions occur at the anterior inferior iliac spine, followed by the anterior superior iliac spine. Avulsion of the ossification center of the iliac crest is the least common type of avulsion fracture in this spectrum.

White et al. identified two types of avulsions at the anterior superior iliac spine. Type 1 involves the insertion of the sartorius and results in anterior displacement of a small fragment, while type 2 involves the insertion of the tensor fasciae latae and results in lateral displacement of a larger fragment [1].

In this case, the ossification center’s avulsion affected the insertion of the tensor fasciae latae and the gluteal fascia on the iliac crest, with the anterior superior iliac spine almost unaffected. The lateral displacement of the avulsion suggests a different origin from the more common avulsions of the iliac crest related to the action of the abdominal oblique and transverse muscles.

Injuries involving pelvic apophyseal avulsions are often misdiagnosed or confused with tendinopathies or muscle tears.

Radiographs can be misleading due to the demonstration of non-mature extraosseous sclerotic changes, which may be misinterpreted as signs of a tumor or infection, especially if the history of initial trauma or athletic overexertion is unknown.
